# Hallmarks of aging in systemic sclerosis: implications for pathogenesis, multimorbidity, and therapeutic development

**DOI:** 10.1016/j.ero.2026.100256

**Published:** 2026-10-14

**Authors:** Maria Iacovantuono, Amela Hukara, Gesa Marie Sauer, Oliver Distler, Maria Sole Chimenti, Gabriela Kania, Cosimo Bruni, Muriel Elhai

**Affiliations:** 1 Department of Rheumatology, University Hospital Zurich, University of Zurich, Zurich, Switzerland; 2 Rheumatology, Allergology and Clinical Immunology, Department of Systems Medicine, University of Rome Tor Vergata, Rome, Italy

## Abstract

Systemic sclerosis (SSc) is a heterogeneous autoimmune disease characterised by marked clinical variability and a substantial burden of comorbidities, which may reflect underlying biological aging processes. Although chronological age influences disease presentation and prognosis, growing evidence suggests that biological aging processes may contribute directly to disease pathogenesis. Hallmarks of aging, including chronic low-grade inflammation and immunosenescence, have been identified across tissues affected by SSc and may drive persistent immune activation, vascular injury, defective tissue repair, and progressive fibrosis.

Aging is increasingly recognised as a tissue-specific and multidimensional process rather than a uniform consequence of chronological time. Differential aging trajectories across organs may influence susceptibility to SSc-related damage, shaping heterogeneous clinical phenotypes. Aging-related mechanisms may also affect disease presentation, severity, comorbidities, and outcomes across the life course and contribute to therapeutic challenges in routine care.

In this review, we examine current evidences linking molecular and cellular mechanism of aging to the pathogenesis, clinical manifestations, and outcomes of SSc. We discuss the implications for comorbidity burden and treatment decision-making. A deeper understanding of aging-related mechanisms may provide a unifying framework for disease heterogeneity and support the development of more individualised management strategies and therapeutic approaches in SSc.

## BACKGROUND

Chronic, low-grade sustained inflammation—generating without an infection (‘sterile’ inflammation)—is known as inflammaging [1]. Together with immunosenescence, it stands at the origin of chronic inflammatory diseases. In recent years, the aging process has been extensively investigated in the context of chronic autoimmune rheumatic diseases. Previous reviews have described the molecular mechanisms of aging in systemic sclerosis (SSc) [2] and in rheumatic diseases more broadly [3]. Although these conditions are not traditionally classified as age-related, increasing evidence highlights the presence of cellular and molecular hallmarks of ageing in their context [3], suggesting that shared biological aging pathways may partly underlie the clinical heterogeneity and comorbidity burden observed in SSc. Building on this framework, this review does not aim to provide an exhaustive overview of ageing-related pathways, but rather to examine how they may contribute to the clinical heterogeneity of SSc. Integrating SSc-specific observations with insights from aging biology thus provides a conceptual framework to investigate whether heterogeneity arises, at least in part, from shared and organ-specific aging trajectories. Finally, we discuss clinical and molecular perspectives on the impact of aging on SSc epidemiology, phenotype, and the burden of comorbidities, with particular attention to how age may inform treatment decision-making in clinical practice. Overall, we are proposing that biological aging may represent a unifying framework linking clinical heterogeneity, organ-specific disease manifestations, comorbidity burden, and differential outcomes in SSc.

## PATHOGENESIS

### Linking aging to disease heterogeneity in systemic sclerosis

Clinical observations suggest that chronological aging alone cannot fully explain SSc-related disease manifestations. Certain features are more prevalent in younger patients, while others do not show a simple increase in severity with age [[4], [5], [6]]. Although age influences both clinical manifestations and the prevalence of specific organ involvements, its relationship with disease expression has only been partly clarified [[7], [8], [9], [10]]. For instance, a large German registry study by Moinzadeh et al [8] demonstrated that patients with disease onset after 60 years were more likely to present with limited cutaneous SSc, exhibited a higher prevalence of pulmonary arterial hypertension (PAH), and a lower frequency of digital ulcers (DUs), whereas also exhibiting a more rapidly progressive course across organ systems. Similarly, data from the Renji Scleroderma Longitudinal Cohort showed that age at onset is associated with distinct organ-specific risk profiles, with early-onset patients showing increased skin and lung involvement, whereas late-onset patients with higher risks of cardiopulmonary, gastrointestinal, and joint complications. Notably, proteomic analyses revealed distinct molecular signatures across age groups, suggesting that these phenotypic differences may reflect underlying biological variations [11]. Although these findings consistently demonstrate clear age-related differences in clinical phenotype, they do not clarify the underlying biological mechanisms behind this variability. Importantly, most existing studies rely on chronological age or subtype-associated markers rather than directly assessing biological aging processes [7,8,10]. As a result, the mechanistic link between aging and disease heterogeneity remains incompletely defined. Although this concept has not been systematically explored in SSc, emerging evidence suggests the involvement of ageing-related pathways.

### The concept of nonlinear aging and windows of vulnerability

Building on the concept that biological aging trajectories may contribute to disease heterogeneity, emerging evidence suggests that aging is not a linear process but occurs in distinct phases characterised by coordinated molecular changes. A recent multiomics study identified 2 distinct inflection points in human aging, occurring at approximately 44 and 60 years of age, respectively, which are associated with increased disease susceptibility [12]. The earlier transition is associated with alterations in lipid metabolism and cardiovascular disease (CVD), whereas the later transition is associated with changes in immune regulation and metabolic pathways, reflecting coordinated shifts across multiple molecular pathways. These transitions may define periods of increased biological vulnerability, during which organisms become more susceptible to disease onset. Notably, these age-related shifts overlap with reported peaks in SSc incidence [13], suggesting that SSc onset may coincide with specific phases of biological aging. This supports the concept of a ‘window of vulnerability’, in which converging age-related molecular changes increases susceptibility to disease-triggering events. Several biological systems undergoing age-related changes—including extracellular matrix remodelling, immune regulation, and vascular pathways—are also central to SSc pathophysiology [8,14], potentially contributing to disease susceptibility rather than representing a direct causal relationship. Importantly, the occurrence of early-onset in childhood, with first Raynaud’s phenomenon and first non-Raynaud’s phenomenon at a mean age of approximately 8 to 10 years [15,16], indicates that chronological age alone may not fully account for disease initiation. Instead, interindividual differences in biological aging trajectories may determine when such vulnerability windows emerge, supporting the notion that accelerated or dysregulated aging processes may contribute to disease susceptibility in a subset of patients [[17], [18], [19]]. Among the biological processes that undergo pronounced age-related changes during these phases, whether these biological transitions are causally linked to disease onset remains unknown.

### Inflammation as a central link in shared aging mechanisms in SSc

Chronic inflammation represents a key mechanism through which aging contributes to the development and progression of autoimmune diseases, including SSc. As a multisystem disorder with broad organ involvement, SSc provides a valuable model disease to examine how shared aging mechanisms manifest across different organs. Despite their distinct clinical presentations, these organs share some common age-related biological alterations, including inflammaging, immune dysregulation, cellular senescence, and aberrant tissue remodelling [20]. Inflammaging, the hallmark of biological aging, is characterised by persistent low-grade inflammation [1] and increased circulating levels of proinflammatory mediators such as interleukin (IL)-6, C-reactive protein (CRP), IL-1β, and tumor necrosis factor [21]. Consistent with this, systemic inflammation has been linked to both organ involvement and mortality in SSc [22]. Elevated or persistently increased CRP levels have emerged as 1 of the most robust prognostic indicators across multiple cohorts [[23], [24], [25]]. Collectively, these findings highlight a role of systemic inflammation in SSc in driving organ involvement and disease progression.

From a molecular perspective, 1 of the major drivers of chronic inflammation is the accumulation of senescent cells. Although these cells have permanently exited the cell cycle, they remain metabolically active and secrete a range of proinflammatory mediators, collectively known as the senescence-associated secretory phenotype, reinforcing inflammation and tissue damage [20]. Given the evidence for cellular senescence in SSc, particularly in immune cells, fibroblasts, and endothelial cells [26,27], this phenotype may represent a key mechanism linking aging-related processes to inflammation, fibrosis, and vasculopathy. At a systemic level, inflammaging and immune dysregulation are closely linked to hematopoietic stem cell aging in the bone marrow, a process underlying immunosenescence [20]. The ‘myeloid bias’, typically seen in aging bone marrow, describes the increased production of proinflammatory myeloid cells at the expense of adaptive immunity. In SSc, this includes the expansion of EGR1⁺ CD14⁺ monocytes, which activate nuclear factor kappa B signalling and contribute to tissue damage [28]. Emerging single-cell and spatial transcriptomic studies further highlight immune alterations in different SSc affected tissues. In SSc skin, proinflammatory vascular niches enriched with macrophages, dendritic cells, and activated fibroblasts point to critical immune-stromal interactions in early disease [29]. In the lung, aging is typically associated with impaired macrophage function and reduced clearance capacity [30,31]. In contrast, SSc lungs exhibit the accumulation of pathogenic macrophage subsets, including SPP1⁺ macrophages, which actively promote fibrotic progression [32]. Adaptive immunity is likewise profoundly altered. Aging leads to a decline in naïve T cells and accumulation of memory T cells [33]. This is accompanied by an expansion of interferon‑γ–driven CD8⁺ effector T cells in blood and lung tissue, as well as CXCL13⁺ T cells in the skin, which may enhance local B-cell activation and perpetuate autoimmunity [28]. Together, these findings demonstrate that age-related inflammatory and immune alterations closely overlap with SSc immunopathology and may contribute to systemic inflammation and organ involvement. Although these observations establish inflammation and immune dysregulation as shared systemic drivers, tissue-specific signatures are already evident, highlighting the difficulty of clearly separating systemic and organ-specific processes. However, these mechanisms alone do not fully explain the marked heterogeneity in organ involvement observed across patients with SSc. Systemic processes such as inflammaging and immunosenescence do not act uniformly across tissues, but instead interact with organ-specific cellular composition, regenerative capacity, and microenvironmental context [20], resulting in distinct pathological outcomes depending on the tissue. This suggests that SSc heterogeneity may arise from the interplay between shared aging mechanisms and tissue-intrinsic vulnerabilities, whereby each organ translates systemic inflammatory and senescence signals into a unique pattern of dysfunction.

### Organ-specific aging mechanisms as a driver of SSc heterogeneity

Although the fundamental drivers of aging are systemic, the mode of functional decline varies considerably across organs, reflecting distinct cellular architecture and regenerative potential [20]. In SSc, these organ-specific aging patterns may intersect with disease-related processes, thereby contributing to the marked disease heterogeneity in clinical presentation.

In the lung, aging is closely linked to dysfunction of alveolar epithelial type II (AT2) cells, which exhibit impaired self-renewal and differentiation capabilities. This is supported by recent evidence demonstrating increased senescence markers and activation of aging-related pathways in SSc-interstitial lung disease (ILD), including enrichment of epithelial and fibroblast senescence signatures and telomere shortening in AT2 cells [34]. Together, these findings support a role for impaired epithelial repair and cellular aging in the development and progression of lung fibrosis. Pulmonary age has been identified as the strongest predictor of mortality [19], aligning with ILD being the leading cause of SSc-related death [35]. Evidence from structural equation modelling further suggests that organ ageing occurs within interconnected networks, with the lung potentially influencing aging trajectories in other systems via nervous, circulatory, and lymphatic networks [19,36].

At the cardiovascular level, aging is characterised by cardiomyocyte hypertrophy, inflammation, and fibrosis. Increased metabolic demand in enlarged cardiomyocytes promotes hypoxia and oxidative stress, leading to the release of proinflammatory mediators and immune cell recruitment [37]. As cardiomyocytes have limited regenerative capacity, tissue damage is substituted by fibrotic scar formation rather than functional repair [38]. Data on cardiac ageing mechanisms in SSc remain limited, partly due to challenges in tissue accessibility. However, experimental studies suggest a role for macrophages in cardiac remodelling, with evidence from a mouse model of bleomycin-induced skin fibrosis and *in vitro* studies showing that SSc macrophages induce profibrotic gene expression in fibroblasts [39]. These findings highlight the need for further research into ageing-related pathways in cardiac involvement in SSc.

The skin shows, as well, how aging-related processes may be translated into hallmark pathological features of SSc, namely fibrosis and vasculopathy. Senescent fibroblasts remain metabolically active and secrete extracellular vesicles and proinflammatory mediators that influence the surrounding tissue environment [40]. Furthermore, a disease-specific *COL8A1/ACTA2⁺* myofibroblast population displays a strong senescence signature and is associated with extracellular matrix expansion [41]. In SSc, human dermal fibroblast-derived vesicles can activate macrophages with reciprocal activation and extracellular matrix deposition [42]. In parallel, aging skin is associated with characteristic changes in cutaneous T-cell populations, including an increase in CD8⁺T cells [43], higher numbers of regulatory T cells [44], and elevated expression of inhibitory receptors such as programmed cell death protein 1 [45], contributing to a low-grade inflammatory but functionally suppressed immune environment. Several of these features partially overlap with observations in SSc skin, such as CD8⁺ T cells being predominant in the early stage of SSc compared with CD4^+^ T cells, whereas more CD4^+^ lymphocytes are found in late-stage disease [46,47]. However, in SSc, these alterations are amplified and accompanied by disease-specific features, including highly activated and profibrotic T-cell subsets as well as impaired immune regulation [[48], [49], [50], [51], [52]]. These findings support the notion that aging-related immune alterations may be modified in SSc, resulting in persistent activation and fibrotic tissue remodelling.

In addition, endothelial dysfunction could represent a critical link between aging-associated processes and SSc pathology. Endothelial dysfunction in SSc skin is characterised by enhanced endothelial-to-mesenchymal transition (EndMT), a process that contributes to vascular remodelling and fibrotic progression [53]. Notably, recent studies have demonstrated that EndMT is significantly increased in SSc skin compared with healthy controls and is positively associated with fibroblast senescence and CCN2 expression, supporting a mechanistic link between vascular remodelling, cellular senescence, and fibrosis [54]. Together, these findings suggest that age-related mechanisms are not simply mirrored in SSc skin but are transformed into disease-specific processes that drive progressive tissue fibrosis and vascular injury.

While systemic processes such as inflammaging and cellular senescence provide a common biological framework, tissue-intrinsic properties determine how these shared mechanisms are translated into organ-specific pathology as observed for SSc. As a result, common aging-associated pathways can lead to distinct outcomes across tissues, including impaired regeneration, fibrosis, and vasculopathy. Importantly, although age-related mechanisms have been described in SSc, an integrated view linking biological aging trajectories to specific patterns of organ involvement is still lacking. Future studies should therefore focus on how aging contributes to disease heterogeneity in SSc.

## SSc EPIDEMIOLOGY

SSc can occur at any age, although most patients develop the disease between 40 and 50 years of age [55]. Clinical data from the German Network for Systemic Scleroderma registry, including over 3000 patients, showed that approximately 24.5% of patients develop their first non-Raynaud’s manifestation before the age of 40, whereas 22.5% present it after the age of 60 [8]. No substantial differences have been observed in the female-to-male ratio in SSc, ranging from 8:1 before 30 years of age to 5:1 after 60 years [56].

When stratifying patient populations according to age at disease onset, some discrepancies emerge. Most studies have defined late-onset SSc as an age at first non-Raynaud manifestation of ≥60 years [7,8,[57], [58], [59]]. Other studies adopted higher thresholds, defining late-onset SSc occurring after the age of 65 years [9] or 70 years [10]. This heterogeneity, together with the progressive increase in life expectancy, raises the need for a standardised and biologically meaningful age cut-off to facilitate patient stratification in future clinical trials.

## CLINICAL MANIFESTATIONS

A deep characterisation of aging-related effects on clinical manifestations is crucial to improve risk stratification, tailor therapeutic strategies, and optimise long-term outcomes. Current guidelines provide little guidance on whether and how age should be considered in SSc management and treatment; nevertheless, both age at disease onset and aging during the disease course may influence clinical presentation and outcomes. Additionally, age-related comorbidities can further complicate the picture of patients with SSc.

### Age at disease onset

Some studies compared early- vs late-onset SSc, highlighting differences in clinical manifestations and outcomes. Despite the limited sensitivity of the preliminary American College of Rheumatology/EUropean League Against Rheumatism (ACR/EULAR) 2007 classification criteria for juvenile SSc (jSSc) [60], this form of the disease differs markedly from adult-onset SSc: the diffuse cutaneous subset is more frequent in jSSc than in adult-onset SSc and the prevalence of anticentromere antibodies (ACA) is lower in jSSc, whereas antibodies associated with overlap syndromes (eg anti-U1 ribonucleoprotein and Pm/Scl) are more frequently observed. In addition, musculoskeletal manifestations are more common in jSSc, whereas severe visceral organ involvement, such as ILD, PAH, and scleroderma renal crisis (SRC), is observed less frequently [61,62]. At the other end of the age spectrum, late-onset SSc is characterised by distinct patterns of organ involvement, antibody profiles, and disease course. In particular, PAH occurs more frequently in these patients [7,63], whereas peripheral vascular manifestations, including Raynaud’s phenomenon and DUs, are less common [57]. In addition, ACA antibodies are more prevalent, and the cutaneous involvement tends to be milder [10]. Compared with earlier-onset disease, it is associated with worse survival [8,10,59]. However, when mortality is assessed relative to the age-, sex-, and calendar year-matched general population, standardised mortality ratios (SMRs) are higher among elderly patients with disease onset before 70 years of age, suggesting a greater disease-specific contribution to mortality in younger patients [10].

### Disease manifestations according to age

To date, few studies have described a significant association between aging and changes in organ involvement during the disease course. Among the available data, the most consistent evidence relates to cardiac involvement. Age-related cardiovascular changes have been shown to overlap with primary cardiac involvement, contributing to more severe cardiac phenotypes in older patients. Allanore et al [64] described older age associated with left ventricular dysfunction on transthoracic echocardiography, and Carreira et al [7] additionally found higher estimated pulmonary systolic pressure on echocardiography and cardiac conduction blocks on electrocardiogram. A similar association was observed for ILD, where aging was linked to its development. Older age has been identified as a risk factor for incident ILD in patients without prior lung involvement [65], and age has also been incorporated as a variable within the ILD-RISC score, a screening algorithm for prescribing high resolution computed tomography in SSc [66]. Not only does aging influence the occurrence of lung involvement but also its progressive functional decline. Older age was identified as a predictor of lung function decline [67,68]. Data on the effects of aging on skin, gastrointestinal, and musculoskeletal involvement remain limited. Age-related decline in kidney function is well recognised, but whether aging also contributes to the development or SRC remains unclear [59]. Available data suggest that renal dysfunction is relatively common in patients with SSc, making it difficult to determine the extent to which renal impairment may be attributable to SSc itself rather than to age-related changes or coexisting comorbidities. Importantly, older patients are more likely to have pre-existing reductions in renal function and age-related comorbidities affecting the kidney, which may complicate the interpretation of renal dysfunction and the distinction between chronic age-related impairment and SSc-related renal involvement [69]. Therefore, age-related decline in renal function and age-associated comorbidities should be considered when interpreting renal impairment in SSc, particularly when distinguishing SSc-related renal involvement from chronic kidney disease attributable to other factors. A higher prevalence of limited cutaneous SSc has been observed in older patients [7], along with an increased occurrence of progressive calcinosis cutis [70], and a greater burden of skin itchiness [71].

### Aging-related comorbidities

SSc is associated with reduced survival, and despite the substantial proportion of deaths directly attributable to the disease itself, mortality can also be influenced by comorbidities [7,35,[72], [73], [74]].

The most frequently reported comorbidities include CVD, infections, cancer, musculoskeletal disorders, and reproductive disorders [72,74,75]. Importantly, age-related comorbidities in SSc are of particular interest. Despite the challenge of distinguishing true comorbidities from SSc-related organ involvement—and their increased burden with aging—evidence suggests an interplay between SSc complications and comorbidities, which may contribute to worse clinical outcomes. The clinical heterogeneity of SSc and its bidirectional interaction with comorbidities may amplify tissue damage and worsen outcomes [75]. Nevertheless, both comorbidity burden and older age independently contribute to poorer prognosis in elderly patients [10], highlighting the need for comprehensive risk assessment and preventive strategies across all age groups.

#### Cardiovascular disease

Like other autoimmune rheumatic diseases, SSc is linked to accelerated atherosclerosis [76,77]. Although microvasculopathy is among the hallmarks of SSc, macrovascular involvement is also more prevalent in SSc than controls [78].

A recent meta-analysis identified cerebrovascular and CVD as major contributors to morbidity and mortality in SSc. Compared with the general population, pooled hazard ratios (HRs) were 1.64 (95% CI, 1.35-2.00) for stroke and 2.12 (95% CI, 1.36-3.30) for CVD [79]. Although some studies suggest a higher frequency of cardiac involvement in older patients [8,80], others do not confirm an age-dependent relationship. A Danish nationwide cohort including 1569 patients with SSc found a higher risk of heart failure in patients ≤55 years than those >55 (HR, 4.14 [95% CI, 2.54-6.74] vs 2.74 [95% CI, 1.98-3.78]). Similarly, secondary cardiovascular outcomes, including arrhythmias, conduction abnormalities, venous thromboembolism, ischemic stroke, and myocardial infarction, appeared largely age-independent [6]. Ischemic stroke was indeed found nearly 3-fold higher in patients <50 years (HR, 2.79; 95% CI, 1.38-5.63) compared with the general population [4].

#### Infections

Infection ranks among the top-5 causes of death in the European Scleroderma Trials and Research group (EUSTAR) database [35,80], with respiratory, skin/soft tissue, gastrointestinal, and urinary tract systems being the most common causes of hospital admission [81]. An increased risk of both bacterial and viral infections has been consistently demonstrated in SSc cohorts compared with the general population, particularly at older age [82,83]. This excess risk likely reflects dysregulation, age-related organ decline, and immunosuppressive therapies [82,83].

#### Cancers

Despite heterogeneity across studies due to differences in sample size, follow-up duration, and study design, multiple cohorts consistently demonstrate an association between older age at SSc onset and cancer development [[84], [85], [86], [87]], possibly reflecting mechanisms linked to chronic inflammation and biological aging. In particular, SSc onset ≥70 years has been strongly associated with subsequent cancer risk [10]. A meta-analysis of 524 SSc-related cancers reported a 75% higher risk compared with the general population (Relative risk [RR], 1.75; 95% CI, 1.41-2.18) [88], along with increased mortality [73,80]. In an Australian cohort of 1727 patients, cancer-related mortality was markedly elevated with a SMR of 3.13 (95% CI, 2.39-4.02) compared with patients with SSc without cancer (SMR, 2.19 [95% CI, 1.87-2.54]) [85]. Age also interacts with immunological factors relevant to malignancy, as shown in the Johns Hopkins Scleroderma Center cohort. Older age predicted a shorter interval between SSc onset and cancer in patients with autoantibody profiles other than RNA polymerase III, whereas their positivity conferred a cancer risk largely independent of aging [89].

#### Osteoporosis and sarcopenia

Patients with SSc are at increased risk of osteoporosis and fragility fractures [5,90,91]. A meta-analysis including 1839 patients reported a pooled prevalence of osteoporosis of 27% (95% CI, 24-31), with aging, menopause, and longer disease duration as major risk factors [91]. Although the link with aging is expected, fractures may occur earlier during life in patients with SSc. A nationwide Taiwanese study showed younger age at hip fracture in SSc compared with controls (67.2 vs 75.2 years), and an increased risk of vertebral fractures (Incidence rate ratio [IRR], 1.75; 95% CI, 1.28-2.35) [5]. Similarly, reduced bone mineral density has been observed in younger women (mean age 35 years), suggesting that bone loss may be present early in the disease course rather than being limited to menopause [92].

Sarcopenia, defined as the age-related decline in skeletal muscle mass, strength and function [93], is more prevalent in SSc than in the general population, contributing to worse disease outcomes [94]. Although its prevalence typically increases with aging, the association is debated, as disease-specific factors—such as longer disease duration, oesophageal involvement, skin fibrosis, and systemic inflammation—may contribute to an earlier onset of muscle loss [95,96]. Consistently, the coexistence of chronic low-grade inflammation related both to aging and to SSc may further accelerate musculoskeletal decline [96].

#### Reproductive health

An increased prevalence of premature menopause has been observed in SSc [97,98], particularly in patients with early disease onset [99]. This likely reflects SSc pathological ovarian dysfunction, including vascular abnormalities [100,101]. Indeed, early and more severe disease onset has been linked to reduced follicular quantity and quality [100]. An increased risk of premature ovarian insufficiency has also been reported, especially between 30 and 40 years (HR, 1.8; 95% CI, 1.2-2.8) [102], possibly driven by ovarian fibrosis and extracellular matrix deposition [103].

### Frailty

Frailty is characterised by reduced physiological reserve, lean body mass, muscle strength, and physical activity [104], and is associated with an increased risk of mortality [105]. In patients with SSc, frailty is recognised as a distinct clinical syndrome that should be considered separately from multimorbidity. Although it is strongly associated with a greater burden of comorbidities, the 2 conditions are not synonymous. While frail individuals are often multimorbid, the opposite is not necessarily true [106]. Furthermore, frailty is no longer regarded as an inevitable consequence of aging but rather as a dynamic and potentially reversible condition that may improve over the course of the disease [107]. Nevertheless, advanced age remains a significant risk factor for frailty, as demonstrated in a large cohort study including 1703 patients with SSc [108].

### Aging-related prognosis

Beyond the higher incidence of comorbidities, aging in scleroderma has also been strongly associated with increased mortality. Both the scleroderma mortality p eustar and the Risk Score for Early Mortality to Stratify for Intensive SSc Therapy (RESIST) include age in their prediction models for mortality, weighting its impact on prognosis alongside distinct clinical characteristics [35,109].

## TREATMENTS

### Current treatment recommendations

Whether chronic inflammation and vasculopathy drive accelerated atherosclerosis and thrombosis independently of age remains unclear, warranting close monitoring. As elderly people more frequently develop CVD, comorbidities, and related complications, treatment decisions require careful evaluation to ensure tailored and safe approaches. Current therapeutic strategies rely on 3 main drug categories, namely immunosuppressant, vasoactive, and antifibrotic agents [110]. However, there are currently no treatment decision-making strategies based on age-specific parameters. Apart from some caution recommended for the management of PAH in patients with CVD comorbidities [111]—which are more common in older individuals—recommendations largely support a standardised approach based on disease manifestations and severity rather than age, as outlined in the different guidelines [[112], [113], [114], [115]]. Similarly, physical and occupational therapy included among the nonpharmacological interventions endorsed by EULAR remain broadly defined across all age-adapted subgroups [116]. In addition, SSc-specific disease monitoring and screening tools are lacking. Thus, the use of CVD risk prediction tools available for the general population is currently advised by EULAR [117]. Current recommendations also emphasise infection risk prevention, including prophylaxis and vaccination as part of routine care, although age-specific guidance remains limited [118,119]. Although vaccination is broadly recommended, its effectiveness may be reduced in older adults due to immunosenescence [120]. This, together with modifiable factors such as nutritional deficiencies and polypharmacy, further complicates personalised prevention [121]. Finally, given the overall increased malignancy risk in SSc, heightened vigilance is warranted across the entire SSc population, particularly among elderly patients [122].

### Age gap between clinical trials and real-world data

As no specific treatment recommendations currently exist for elderly patients with SSc, this population remains underrepresented in randomised controlled trials (RCTs) [123]. Among the largest SSc RCTs, mean participant age ranged from 40 to 50 years (Table) [[124], [125], [126], [127], [128], [129], [130], [131], [132], [133], [134], [135], [136], [137],[138], [139], [140], [141]], which may limit the applicability of trial findings to real-world clinical practice—particularly for older patients. Age-based exclusion criteria further compound this issue: the Autologous Stem Cell Transplantation International Scleroderma (ASTIS) trial excluded patients >65 years [129], and the Scleroderma: Cyclophosphamide or Transplantation (SCOT)trial those >69 [134]. Similarly, restrictions tied to disease duration—such as enrolment within 5 to 7 year of the first non-Raynaud’s phenomenon—create age-dependent barriers that systematically exclude both patients with late-onset SSc and incident SSc presenting later in life.

**Table:** Mean age of included patients and age ranges defined by inclusion criteria in clinical trials of systemic sclerosis

Study (ref.)	Treatment	Duration	No. of participants	Age inclusion criteria (y)	Mean age	Disease duration	Primary outcome
[124]	MTX i.m. vs placebo	48 wk	29	≥16	54	<3 y from the first signs of skin thickening	TSS or VAS improvement (total skin score), pDLCO% improvement ≥15%
BREATHE-1 [125]	Bosentan vs placebo	16 wk	213	No age restrictions	48.3	/	Change in 6 MWT at 16 wk
SLS I [126]	CYC oral vs placebo	12 mo	158	No age restriction	47.9	Onset of the first n-RP ≤ 7 y	Change in pFVC% at 12 mo
ASSIST [127]	HSCT vs CYC	24 mo	19	<60	44.5	/	Change in mRSS or pFVC% at 12 mo
SERAPHIN [128]	Macitentan vs placebo	36 mo	742	No age restrictions	45.6	/	Time to first confirmed morbidity or mortality event
ASTIS [129]	HSCT vs CYC i.v.	24 mo	156	18-65	44	≤ 4 y	Event-free survival at 4 y follow-up
GRIPHON [130]	Selexipag vs placebo	event-driven trial	1156	18-75	48.1	/	First morbidity event or death
FaSScinate [131]	Tocilizumab vs placebo	48 wk	87	≥18	49.5	Onset of the first n-RP ≤ 60 mo	Change in mRSS at 24 wk AEs and SAEs at 48 wk
SLSII [132]	CYC vs MMF	24 mo	142	18-75	52.1	Onset of first n-RP ≤ 7 y	Change in pFVC% at 12 mo
PATENT-1 PATENT-2 [133]	Riociguat vs placebo	12 wk	111	No age restrictions	57	/	Change in 6 MWD at 12 wk
SCOT [134]	HSCT vs CYC	54 mo	75	18-69	46	Maximum disease duration 5 y	Global rank composite score at 54 mo
SENSCIS [135]	NTB vs placebo	52 wk	576	≥18	54	Onset of first n-RP ≤ 7 y	Change in pFVC% at 52 wk
FocuSSced [136]	Tocilizumab vs placebo	48 wk	210	No age restriction	48.2	Onset of the first n-RP ≤ 60 mo	Change in mRSS at 48 wk
ASSET [137]	Abatacept vs placebo	12 mo	88	≥18	49	Onset of the first n-RP ≤ 36 mo	Change in mRSS at 12 mo
DesiReS [142]	RTX vs placebo	24 wk	56	20-79	48.7	/	Change in mRSS at 48 wk
STELLAR [138]	Sotatercept vs placebo	24 wk	106	≥18	48.3	/	Change in 6 MWD at 24 wk
SENSCIS ON [139]	Continued NTB vs initiated NTB	52 wk	444	≥18	55.1	Onset of first n-RP ≤7 y	Change in pFVC% at 52 wk
RECITAL [140]	RTX vs CYC i.v.	48 wk	145	18-80	56.6	/	Change in pFVC% at 48 wk
ReSScue [141]	ACHIM vs placebo	20 wk	67	18-85	59	/	Change in UCLA GIT score at 12 wk

ACHIM, anaerobic cultivated human intestinal microbiome; CYC, cyclophosphamide; HSCT, haematopoietic stem cell transplantation; i.m., intramuscular; i.v., intraenous; mRSS, modified Rodnan Skin Score; MTX, methotrexate; n-RP, non-Raynaud’s phenomenon; NTB, nintedanib; pDLCO%, predicted diffusing capacity for carbon monoxide; pFVC%, predicted forced vital capacity; RTX, rituximab; TSS, total skin score; UCLA GIT, University of California, Los Angeles Scleroderma Clinical Trial Consortium Gastrointestinal Tract 2.0; VAS, visual analogue scale; 6 MWD, 6-minute walking distance.

This is especially concerning given that real-world data indicate a substantial burden of SSc among patients aged 70 to 84 years [143]. Collectively, these data highlight a significant gap between trial populations and the broader patient population encountered in clinical practice, with older patients representing a prevalent yet consistently underrepresented group.

### Treatment safety concerns

To date, few real-life studies examined age-related differences in prescription patterns and safety among patients with SSc and available evidence highlights substantial missing data in age-stratified treatment decision-making in SSc.

#### Evidence from clinical trials and real-world data in aging

RCTs rarely addressed medication safety from an age-specific perspective. Most include age- and sex-matched populations - with "sex" referring to biological sex - providing limited evidence on age-related effectiveness or safety, and no specific RCT has been conducted in juvenile jSSc because of its low incidence and prevalence, and treatment largely relies on extrapolation from adult studies [144]. In contrast, while no effectiveness data are available to date, evidence suggests that aging influences the prescription trends and raises safety concerns. Patients with autoimmune rheumatic diseases are also more likely to receive concomitant therapies such as glucocorticoids, antidiabetic drugs, diuretics, beta-blockers, and lipid-lowering agents and may have an increased risk of ischemic stroke, infections, and poorer survival [4]. Therefore, treatment should be individualised on a case-by-case basis. Vasoactive combination therapy is the mainstay of SSc-associated PAH, but older patients require caution and tighter monitoring due to comorbidities, lower tolerance, higher discontinuation rates, and relevant drug-drug interactions; as a result [145], monotherapy is often preferred [111]. Real-world data indicate undertreatment in the elderly patients, with reduced use of vasoactive therapies in patients with onset ≥70 years [10], consistent with French and German cohorts showing less frequent treatment prescription despite higher PAH prevalence in older patients [8,146]. Notably, in the Australian Scleroderma Cohort, treatment did not differ by comorbidity burden (combination therapy vs monotherapy), but monotherapy was associated with worse survival [147]. Thus, addressing the balance between comorbidity-driven caution and the risk of undertreatment is essential to avoid therapeutic inertia in older patients. Regarding immunosuppressive therapy, EUSTAR registry data showed that age did not influence the initiation of immunosuppressive therapy or ‘antifibrotics’ to treat patients with ILD. Reassuringly, the median age at treatment initiation has increased over time, suggesting a broader use of these therapies in older patients [148]. However, combination therapy remains associated with younger age (OR, 0.968; 95% CI, 0.953-0.985) [148]. Real-world safety evidence remains limited. In a cohort of 545 patients treated with mycophenolate mofetil, older age was associated with a numerical trend towards more adverse events, including gastrointestinal intolerance and severe infections [149]. Similarly, age did not affect rituximab retention in 2 studies [150,151]. Newer therapies also require caution. Janus Kinase inhibitors (JAK) have a limited role in SSc [113], but are increasingly used in practice [152]. However, post-hoc analysis on rheumatoid arthritis patients treated with JAK inhibitors suggests a higher risk of major adverse cardiovascular events in patients with CVD risk factors [153], thus requiring caution in the elderly. Conversely, post-hoc analyses of nintedanib trials [(INBUILD and Safety and Efficacy of Nintedanib in Systemic Sclerosis (SENSCIS)] suggest no major age-related increase in adverse events, although older patients may require closer monitoring [154]. Emerging approaches such as autologous hematopoietic stem cell transplantation and Chimeric Antigen Receptors T cells (CAR-T) appear more dependent on biological rather than chronological age, although eligibility must balance infectious risk under intensive immunosuppression [155]. To date, the use of autologous hematopoietic stem cell transplantation and CAR-T remains limited by the need to balance therapeutic benefit against the infectious risks associated with intensive immunosuppression [155]. In this context, incorporating age into the RESIST score improves early mortality prediction in patients eligible for cellular therapies, supporting a more personalised, risk-adapted treatment approach [109].

## FUTURE PERSPECTIVES

In summary, aging is a key risk factor for age-related diseases (Fig); however, the role of biological aging-related pathways across organ systems remains insufficiently explored in SSc pathogenesis. Future studies leveraging large, longitudinal cohorts with detailed clinical phenotyping are needed to characterise patient-specific aging trajectories at both systemic and organ levels, integrating clinical and molecular data. A better understanding of aging-related mechanisms may help identify novel therapeutic targets and biomarkers, enabling earlier and potentially preventive treatment strategies, as well as more tailored approaches across different age groups. Although some aging-related molecular and clinical characteristics, comorbidities, and related complications may also be relevant to other systemic autoimmune diseases with aging, their impact in SSc deserves specific consideration given its distinctive phenotype combining fibrosis, inflammation and vascular changes. Importantly, as survival continues to improve and the SSc population progressively ages, clinical practice is likely to evolve. Clinicians may increasingly shift from the management of life-threatening acute organ complications towards the recognition and management of age-related chronic disease burden. This transition may require greater emphasis on long-term disease management, preservation of physical function, and management of age-related comorbidities. Collectively, these considerations underscore the importance of integrating aging-related factors into both clinical care and therapeutic development in SSc.

**Figure:**
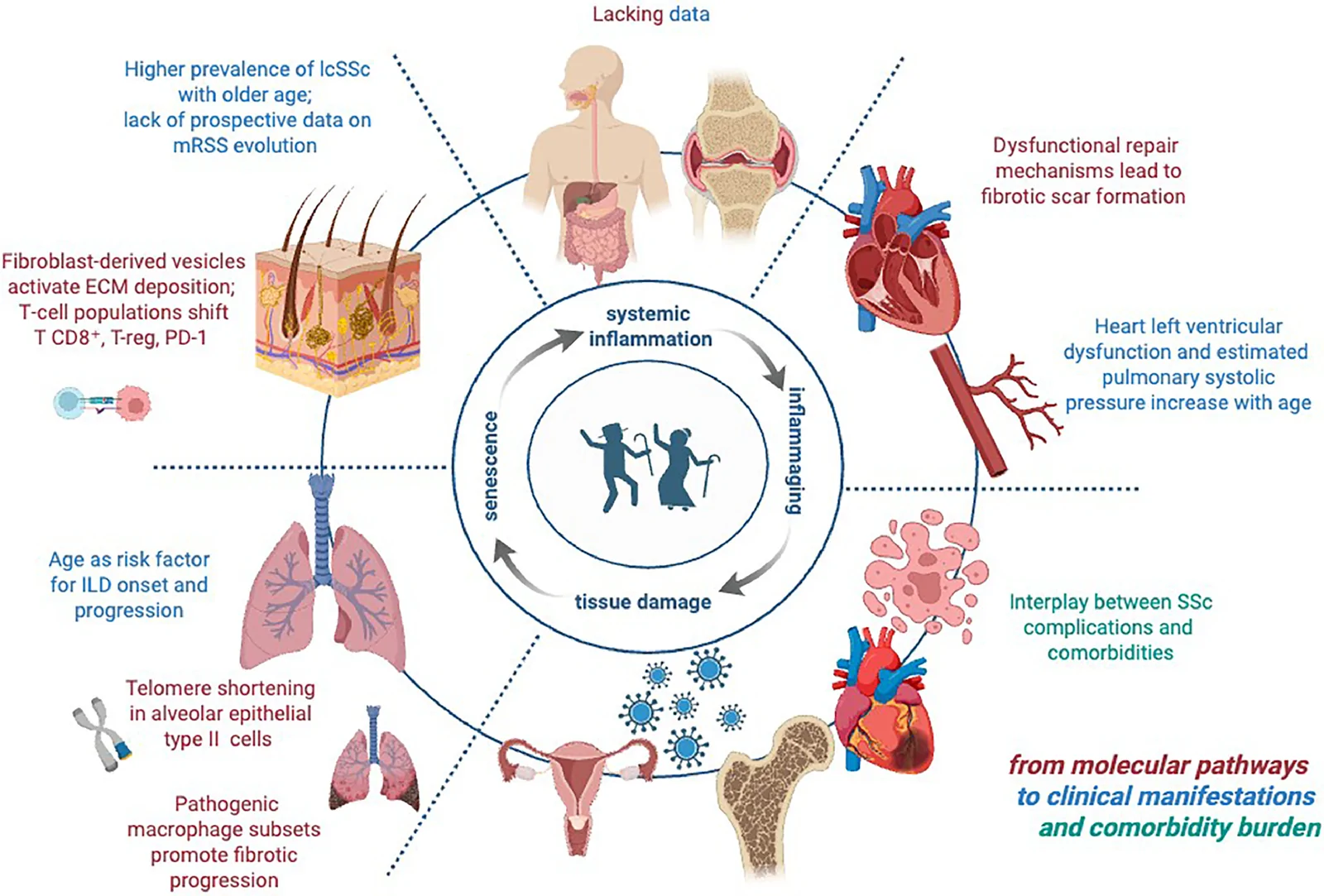
The complex interplay among inflammaging, organ-specific ageing, and comorbidity burden in systemic sclerosis. ECM, extracellular matrix; ILD, interstitial lung disease; lcSSc, limited cutaneous systemic sclerosis; mRSS, modified Rodnan Skin Score; PD-1, programmed cell death protein 1; SSc, systemic sclerosis; T-reg, regulatory T cell.

## Funding

This research did not receive any specific grant from funding agencies in the public, commercial, or not-for-profit sectors.

## CRediT authorship contribution statement

**Maria Iacovantuono:** Writing – review & editing, Writing – original draft, Conceptualization. **Amela Hukara:** Writing – review & editing, Writing – original draft. **Gesa Marie Sauer:** Writing – review & editing, Writing – original draft. **Oliver Distler:** Writing – review & editing, Supervision. **Maria Sole Chimenti:** Writing – review & editing, Supervision, Conceptualization. **Gabriela Kania:** Writing – review & editing, Supervision. **Cosimo Bruni:** Writing – review & editing, Supervision. **Muriel Elhai:** Writing – review & editing, Writing – original draft, Validation, Supervision, Conceptualization.

## Competing interests

MI has received research grant from EULAR long-term research fellowship and EUSTAR visiting fellowship; Congress support from Ely Lilly and Alfasigma; GMS had Congress support from Schweizerische Gesellschaft für Rheumatologie and Boehringer Ingelheim; OD has/had consultancy relationships with and/or has received research funding from and/or has served as a speaker for the following companies in the area of potential treatments for systemic sclerosis and its complications in the last 3 calendar years: 4P-Pharma, AbbVie, Acepodia, Aera, Amgen, AnaMar, Anaveon, Argenx, AstraZeneca, Avalyn, Boehringer Ingelheim, BMS, Calluna, Cantargia, CSL Behring, EMD Serono, Galderma, Fimmcyte, Galapagos, Gossamer, Hemetron, Innovaderm, Kali, Lilly, Mediar, MSD Merck, Nkarta, Novartis, Oorja Bio, Orion, Pliant, Prometheus, Quell, Scleroderma Research Foundation, Skyhawk, Tandem, Topadur, UCB, and Umlaut.bio. Patent issued ‘mir-29 for the treatment of systemic sclerosis’ (US8247389, EP2331143). MSC is a co-founder of CITUS AG; has/had consultancy relationships with AbbVie, Eli Lilly, Johnson and Johnson, Novartis, and UCB; CB has/had consultancy relationships with AbbVie, Boehringer-Ingelheim, and GlaxoSmithKlein; had Congress support from Boehringer-Ingelheim. ME has/had grant/research support from Vontobel Stiftung, Pfizer, Novartis Foundation for Bio-Medical Research, Iten Kohaut Foundation, Kurt und Senta Herrmann Foundation, Foundation for research in Rheumatology (FOREUM), and University of Zurich; Congress support from AstraZeneca; and Speaker and consultancy fees from Boehringer Ingelheim.
